# Protective Effects of Annona Atemoya Extracts on Inflammation, Oxidative Stress, and Renal Function in Cadmium-Induced Nephrotoxicity in Wistar Rats

**DOI:** 10.3390/ph17101393

**Published:** 2024-10-18

**Authors:** Alexandre Coelho Serquiz, Joana de Angelis da Costa Barros Gomes, Naisandra Bezerra da Silva Farias, Denise Mafra, Pietra Maria Pereira de Lima, Daniella de Oliveira Coutinho, Fernanda Priscila Barbosa Ribeiro, Hugo Alexandre de Oliveira Rocha, José Luiz de Brito Alves

**Affiliations:** 1 Department of Nutrition, Health Sciences Center, Federal University of Paraíba, João Pessoa 58051-900, PS, Brazil; alexandreserquiz@gmail.com (A.C.S.); pietrampl20@gmail.com (P.M.P.d.L.); daniella.coutinho@academico.ufpb.br (D.d.O.C.); fernanda.barbosaribeiro@ufpe.br (F.P.B.R.); 2 Laboratory of Biotechnology of Natural Polymers (BIOPOL), Graduate Program of Biochemistry and Molecular Biology, Bioscience Center, Federal University of Rio Grande do Norte—UFRN, Natal 59078-970, RN, Brazil; joannabarros@yahoo.com.br (J.d.A.d.C.B.G.); naisandra@ufrnet.br (N.B.d.S.F.); hugo-alexandre@uol.com.br (H.A.d.O.R.); 3 Graduate Program in Biological Sciences—Physiology, Federal University of Rio de Janeiro (UFRJ), Rio de Janeiro 21941-902, RJ, Brazil; dmafra30@gmail.com

**Keywords:** cadmium, atemoya fruit, inflammation, oxidative stress, nephrotoxicity

## Abstract

Background: Cadmium (Cd), a highly toxic heavy metal from agricultural activities, and its exposure can lead to impaired renal function by increasing reactive oxygen species. The atemoya fruit is known for its high phenolic and antioxidant compounds. This study aimed to evaluate the effects of atemoya extracts on renal function, oxidative stress parameters, and inflammatory biomarkers in a cadmium-induced nephrotoxicity model. Methods: Three aqueous extracts were prepared from different parts of the atemoya fruit: seeds, peel, and pulp. Twenty-five male *Wistar* rats were allocated into four groups: control, seed, peel, and pulp extracts at 2 g/kg for 25 days. All treatment groups administered intraperitoneal injections of cadmium chloride (CdCl_2_) (2 mg/kg) to induce renal damage. Results: The cadmium-treated groups showed decreased creatinine clearance, SOD, CAT, and GPx activities (*p* < 0.05) and increased serum levels of TNF-α and IL-6 compared to the control group (*p* < 0.05). The treatment with seed, peel, and pulp extracts increased creatinine clearance (*p* < 0.05), increased SOD, CAT, and GPx activities (*p* < 0.05), and reduced serum levels of TNF-α and IL-6 compared to the Cd group (*p* < 0.05). Conclusions: This study supports the use of atemoya as a promising candidate for mitigating nephrotoxicity and highlights the importance of its antioxidant and anti-inflammatory properties in renal health.

## 1. Introduction

Kidneys are regulatory organs involved in the body’s homeostasis, responsible for maintaining hydroelectrolytic and acid-base balance, eliminating metabolic wastes, regulating blood pressure, and secreting hormones [1]. The kidney is a sensitive organ to environmental exposure to heavy metals [2]. Environmental pollutants are recognized risk factors for the development of kidney disease, a condition that has become a public health problem [3].

Cadmium (Cd) is a xenobiotic used in industry and agriculture with a recognized nephrotoxic effect [4]. Exposure to Cd occurs through the ingestion of contaminated food and water, as well as the inhalation of dust and cigarette smoke. As a result of this exposure, Cd accumulates in the body’s tissues, with the kidneys being the primary organs affected, leading to renal dysfunction [5].

The Cd accumulates in the proximal tubules, compromising the cellular and functional integrity of these cells [6]. Cd is filtered by pinocytotic vesicles at the border of proximal tubular cells and transported to lysosomes, where the protein is degraded, releasing free Cd^+2^ ions. These ions are then released into the tubular lumen, causing damage to renal tubular cell membranes by reducing cellular respiration and oxidative phosphorylation [7]. This process is primarily driven by increased oxidative stress, which produces superoxide anions and nitric oxide, inhibiting antioxidant enzyme activities and exacerbating kidney damage [8].

Oxidative stress is a primary mechanism leading to kidney damage [9]. Cd disrupts the redox balance by depleting reduced glutathione (GSH) and inhibiting catalase, enzymes responsible for the clearance of reactive oxygen species (ROS). In addition to impairing the activity of antioxidant enzymes, Cd binds to sulfhydryl groups in proteins, increasing ROS production [9]. ROS plays a crucial role in tubulointerstitial fibrosis by activating myofibroblasts, promoting scar tissue formation, and leading to the loss of renal function [10].

Evidence suggests that plant extracts may play a protective role in models of Cd-induced renal injury by reducing oxidative effects on renal cells [3]. The atemoya fruit (*Annona cherimola* Mill. × *Annona squamosa* L.) is a hybrid between the cherimoya (*Annona cherimola* Mill.) and the sugar apple (*Annona squamosa* L.) [11]. Its antioxidant activity is attributed to the presence of phenolic substances, mainly flavonoids, which are bioactive compounds of great scientific interest due to antioxidant, anti-inflammatory, and anticarcinogenic properties [12].

Although an early study has shown that seed extract of atemoya can exert anti-inflammatory and reno-protective effects [13], the antioxidant, anti-inflammatory, and reno-protective effects of different parts of atemoya remain to be investigated. Therefore, this study aimed to analyze the antioxidant, anti-inflammatory, and reno-protective effects of atemoya seed, peel, and pulp extracts in cadmium-induced renal damage.

## 2. Results

Cadmium-exposed rats showed a 55% reduction in body-weight gain compared to controls, and none of the treatments were able to prevent this weight loss (Table 1). Cadmium administration also resulted in a 19% decrease in food consumption, with all treated groups showing a decrease in food consumption. Diuresis showed no significant differences among the cadmium-treated groups. Treatment with extracts from different parts of the atemoya did not prevent cadmium-induced changes in body weight gain and food intake (Table 1).

**Figure 1:**
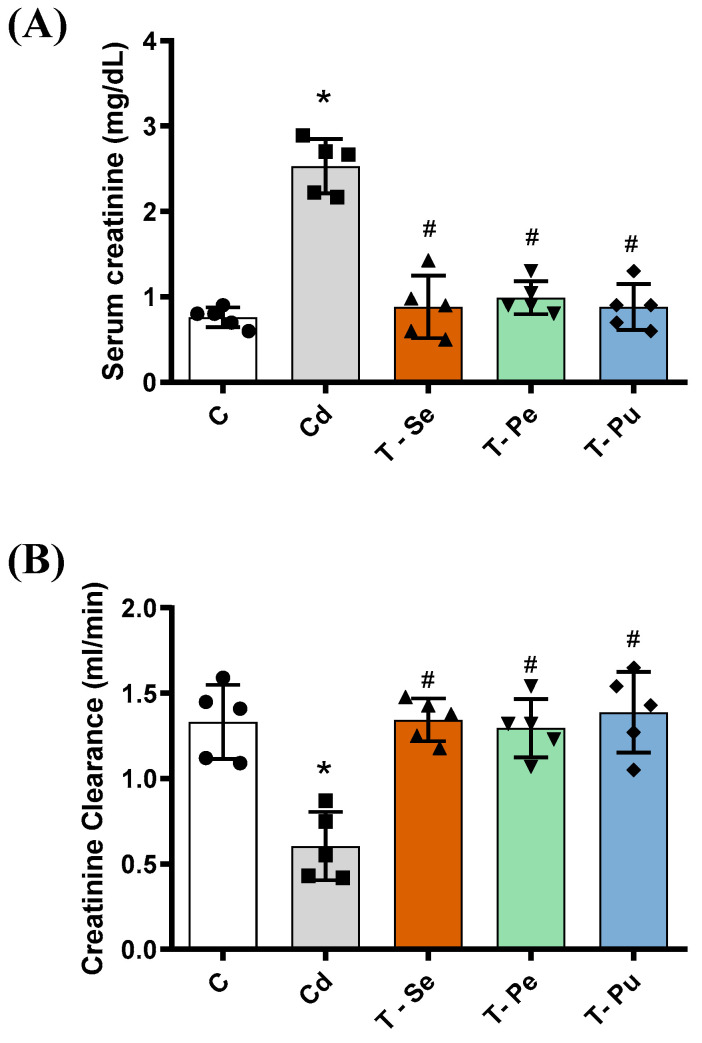
The administration of the peel, seed, and pulp extracts of atemoya improved renal function parameters. group (C) consisted of rats that were not exposed to any treatment (*n* = 5); cadmium group (Cd), the rats received intraperitoneal injections of cadmium (2 mg/kg); T-Se (rats treated with the lyophilized seed extract, *n* = 5); T-Pe (rats treated with the lyophilized bark extract, *n* = 5); T-Pu (rats treated with the lyophilized pulp extract, *n* = 5). All three groups received equal doses of different parts of atemoya (2 g/kg body weight). Administration of seed, peel and pulp extracts of atemoya reduced serum creatinine levels (**A**) and increased creatinine clearance (**B**) in cadmium-induced nephrotoxicity in Wistar rats. Results are expressed as mean ± SEM. The means of the groups were compared by one-way ANOVA followed by Tukey’s test: * *p* < 0.05 versus control group; # *p* < 0.05 versus Cd group.

Rats treated with cadmium exhibited higher serum creatinine levels (33%) and lower creatinine clearance (39%) compared to the control group (*p* < 0.05, Figure 1A,B). The treatment with seed, peel, and pulp extracts of atemoya reduced creatinine and increased creatinine clearance in cadmium-induced nephrotoxicity in *Wistar* rats (*p* < 0.05, Figure 1A,B).

**Figure 2:**
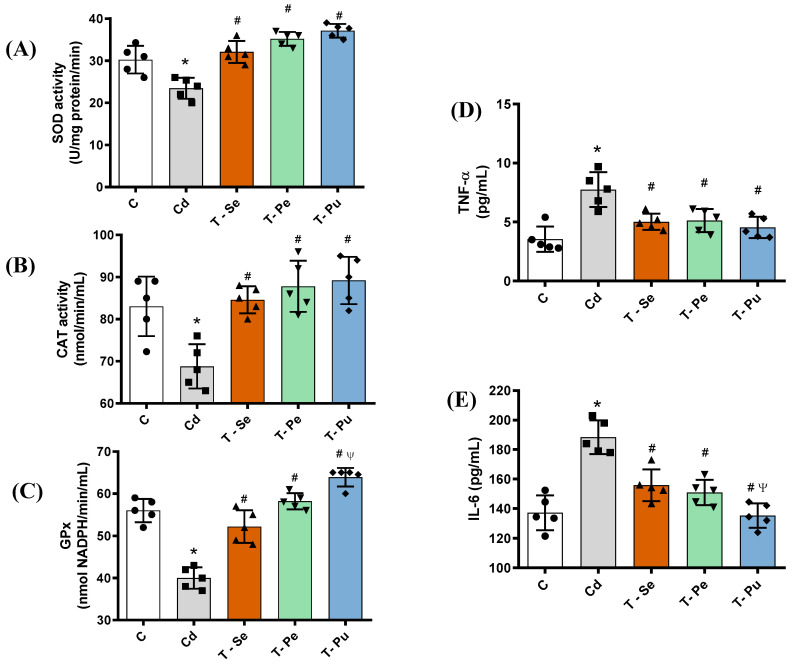
The administration of the peel, seed, and pulp extracts of atemoya increased antioxidant enzyme activities in serum and decreased serum inflammatory biomarkers. Groups: control group (C) consisted of rats that were not exposed to any treatment (*n* = 5); cadmium group (Cd), the rats received intraperitoneal injections of cadmium (2 mg/kg); T-Se (rats treated with the lyophilized seed extract, *n* = 5); T-Pe (rats treated with the lyophilized bark extract, *n* = 5); T-Pu (rats treated with the lyophilized pulp extract, *n* = 5). All three groups received equal doses of different parts of atemoya (2 g/kg body weight). Administration of seed, peel and pulp extracts of atemoya increased serum SOD, CAT and GPx activities (**A**–**C**) and decreased serum levels of TNF-α and IL-6 (**D**,**E**) in cadmium-induced nephrotoxicity in Wistar rats. Results are expressed as mean ± SEM. The means of the groups were compared by one-way analysis of variance followed by Tukey’s test: * *p* < 0.05 vs. control group; # *p* < 0.05 vs. Cd group; Ψ *p* < 0.001 vs. T-Se group.

The cadmium-treated group showed a decrease in catalase (17%), superoxide dismutase (22%), and glutathione peroxidase (29%) (*p* < 0.05, Figure 2A–C). Treatment with seed, peel and pulp extracts of atemoya increased the activities of SOD, CAT and GPX compared to the Cd group (*p* < 0.05, Figure 2A–C). The serum levels of IL-6 and TNF-α were increased in the Cd group compared to the CTL group (*p* < 0.05, Figure 2D,E). Treatment with seed, peel and pulp extracts of atemoya reduced serum levels of IL-6 and TNF-α in cadmium-induced nephrotoxicity in Wistar rats (*p* < 0.05, Figure 2E).

**Figure 3:**
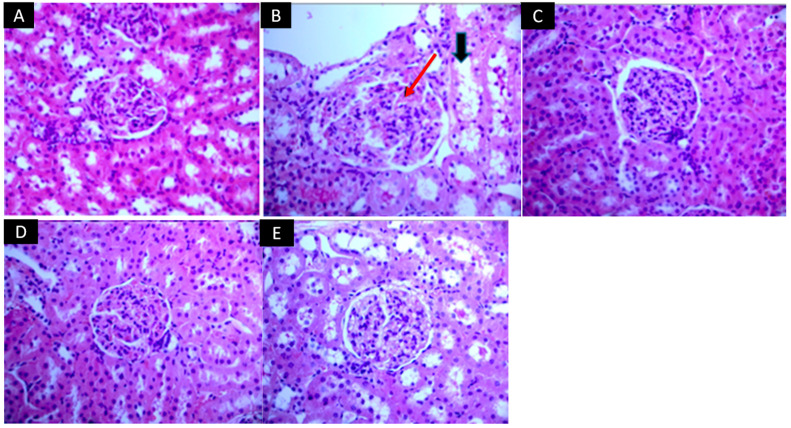
Representative images illustrating the effects peel, seed, and pulp extracts of atemoya in cadmium-induced nephrotoxicity in Wistar rats. Groups: control group (**A**,**C**) consisted of rats that were not exposed to any treatment (*n* = 5); cadmium group (Cd) (**B**), the rats received intraperitoneal injections of cadmium (2 mg/kg); T-Se (**C**) (rats treated with the lyophilized seed extract, *n* = 5); T-Pe (**D**) (rats treated with the lyophilized bark extract, *n* = 5); T-Pu (**E**) (rats treated with the lyophilized pulp extract, *n* = 5). All three groups received equal doses of different parts of atemoya (2 g/kg body weight). Red arrows indicate glomerular hypercellularity and hyperemia. Black arrows indicate tubular dilation.

Renal histology is shown in Figure 3. Cadmium administration (Figure 3B) altered renal morphology and caused histopathological damage. The group treated with cadmium (Cd) showed a pronounced lymphocytic infiltrate around the glomerulus and damage to the brush border of the tubules (indicated by the red arrow). This histological section also shows marked hyperemia within the glomerulus and disorder of the tubular borders with loose nuclei (indicated by the black arrows), which is not observed in the control group (C) (Figure 3A). Figure 3C,D show the T-Se and T-Pe groups, respectively, which presented intact glomeruli and well-defined tubules. In contrast, the T-Pu group shown in Figure 3E exhibited some degree of hyperemia. Table 2 presents the renal histological changes based on a scoring scale. It was observed that cadmium modifies the morphology of tubular cells and promotes inflammation, and that treatments with the peel, seed, and pulp of atemoya can prevent these alterations in nephron morphology.

## 3. Discussion

Exposure to cadmium, even at low levels, can damage various tissues, including the renal, cardiovascular, hepatic, and skeletal systems [9]. In this study, we confirmed that cadmium administration significantly reduces body weight gain and elevates serum creatinine and the inflammatory cytokines IL-6 and TNF-α. It has been demonstrated that treatment with lyophilized extracts of atemoya seeds, peel, and pulp has a nephroprotective effect due to its antioxidant and anti-inflammatory potential, as evidenced by increased levels of all antioxidant enzymes and reduced plasma levels of IL-6 and TNF-α, which are cytokines that rise when renal alterations occur, being involved in several diseases of this organ (acute kidney disease, chronic kidney disease, glomerulonephritis, focal segmental glomerulosclerosis) [14]. Their increased concentration is implicated in the worsening of kidney injury, as they mediate many cytotoxic and pro-inflammatory actions in this organ [15].

Cadmium disrupts tubular membranes, leading to defects in reabsorption and secretion processes, which results in the accumulation of substances such as amino acids, calcium, uric acid, and potassium in the urine [16]. Among these substances, urea and creatinine are commonly used as markers of kidney function, with creatinine clearance being a standard measure of glomerular filtration rate [17]. It has been proposed that cadmium-induced kidney damage is associated with epithelial cell degeneration and hypertrophy, glomerular distortion, and localized hemorrhage within the renal tubular tissues [18,19].

Cadmium affects the entire kidney, particularly the proximal tubules, causing toxic and harmful effects even with relatively low accumulation in the organ [20]. Our findings reveal that the excretion and accumulation of this metal in the kidneys led to structural and functional damage. Increased tubular cell proliferation, inflammation, tubular dilation, epithelial necrosis, hyperemia, and glomerular hypercellularity were observed in the Cd group, while treatment with atemoya extracts did not exhibit significant changes in tubular cell proliferation, epithelial necrosis, hyperemia, or glomerular hypercellularity. Additionally, the treatment promoted a protective effect on renal filtration. No significant differences were found between the groups treated with atemoya seed, peel, and pulp, indicating that all extracts have nephroprotective effects. Other species of Annona, such as squamosa and cherimola, have reported nephroprotective effects against ifosfamide-induced oxidative stress in the kidneys of rats [13].

Studies using Annona atemoya as a treatment for kidney protection are scarce, but it is well established that this fruit contains phytochemical components and phenolic constituents [21]. Like other fruits from the Annonaceae family, atemoya is rich in phenolic compounds [21,22]. Moraes et al. characterized the phenolic compounds of atemoya pulp and seeds through liquid chromatography and identified a high concentration of catechin and epicatechin [22]. In the present study, all parts of the fruit exhibited antioxidant activity, highlighting the overall potential of atemoya as a source of beneficial compounds. This approach also aligns with principles of utilization and sustainability, as it encourages the use of the entire fruit and minimizes waste.

Although the exact mechanisms of action against nephrotoxicity are not yet fully understood, antioxidant and anti-inflammatory activities have been reported in atemoya fruit extracts [21,23]. The nephroprotective effect is likely attributed to phenolic compounds, as they are potent antioxidants. This was observed in a similar study conducted on rats treated with a nephrotoxic drug (ifosfamide), where atemoya was used to attenuate the effects of oxidative stress caused by the drug. The study identified an increase in the activity of antioxidant enzymes glutathione peroxidase and catalase, downregulated the expression of iNOS and NF-kB, as well as the immunohistological expressions of caspase-3 and BAX, and upregulated the expression of Bcl-2, reflecting an improvement in renal function (reduced serum creatinine and urea levels) [13]. Another substance present in atemoya that may be involved in kidney protection, found in higher concentrations in the pulp and peel of the fruit, is gallic acid (3,4-dihydroxybenzoic acid), which has antioxidant and anti-inflammatory effects. Rats that underwent renal ischemia–reperfusion injury and were pretreated with gallic acid showed improvement in renal malondialdehyde levels, serum glutathione, and glutathione peroxidase activity [23]. Thus, the nephroprotective effect may be related to the suppression of inflammation, as evidenced by the reduction in inflammatory markers and the decrease in oxidative stress [24,25].

Antioxidant enzymes play a crucial role in protecting against cellular dysfunction caused by increased free radicals, and their elevated levels are associated with potential renal protection in cases of nephropathy [26]. The present study showed that the groups treated with lyophilized extracts of T-Se (seed), T-Pe (peel), and T-Pu (pulp) had increased antioxidant enzymatic activity compared to the Cd group, with the most notable increase observed in the T-Pu group when compared to the T-Se group. Previous studies have shown that extracts rich in antioxidants or antioxidant molecules reduce Cd-induced oxidative stress in the kidney and other organs by reducing free radicals and increasing antioxidant enzyme activity [27,28,29,30].

The Cd group exhibited significant increases in interleukin-6 (30%) and TNF-α (119%) levels compared to the control group, indicating a probable increase in the inflammatory process. Inflammation in the Cd group is consistent with previous findings [31,32]. In contrast, rats treated with atemoya seed, peel, and pulp extracts showed reduced plasma levels of inflammatory cytokines compared to the group that received only cadmium. A possible explanation for this effect is the reduction in cellular oxidative stress by the polyphenols present in these extracts, leading to reduced stimulation of inflammatory cytokines [33].

Studies have demonstrated that the nephroprotective effects of phenolic compounds, such as those found in atemoya, are strongly linked to their ability to mitigate oxidative stress caused by toxic substances. Our results reinforce this association and highlight how the antioxidant properties of atemoya extracts effectively neutralize oxidative damage and provide renal protection [13,34].

## 4. Methods and Materials

### 4.1. Extract Preparation

Atemoya (*Annona cherimola* Mill. × *A. squamosa* L.) fruit samples were collected in Natal, Rio Grande do Norte, Brazil. The fruit, with the skin intact, was cleaned and sanitized by washing with running water and then immersed in chlorinated water (250 ppm) for 15 min. Afterwards, the fruit was rinsed with potable water to remove chlorine residues, following the Brazilian Sanitary Surveillance legislation (RDC No. 216). After sanitization, the seeds, peel, and pulp were manually extracted and separated. The peels were frozen at −20 °C and thawed only at the time of extraction. The seeds, pulps and peels were dried in a ventilated oven at 60 °C, peeled to remove the tegument, and the cotyledons were ground in an industrial mill until a fine flour was obtained. For the preparation of aqueous extracts, the three parts (peel, seed, and pulp) were individually weighed and then diluted in distilled water in different proportions: 1:1; 1:4; 1:8 and 1:10 (g of fruit extract/mL of distilled water). The dilutions of 1:1 for seeds and pulp and 1:10 for the peel had the better antioxidant capacity in vitro test [35]. The contents of phenolic compounds in dry extracts were 15 mg, 17 mg, and 30 mg in 2 g of extract for the seeds, pulp, and peel, respectively.

After dilution, the extracts were ground for 3 min at 4 °C and then homogenized for 2 h using a magnetic stirrer. The diluted extracts of peel, pulp, and seed were then individually centrifuged at 7500× *g* for 30 min at 4 °C and filtered through filter paper. After filtration, the extracts were lyophilized for 24 h. These components were then separately stored in 50 mL conical tubes at −80 °C until use.

### 4.2. Experimental Animals

The animal experimental protocol was approved by the Animal Experimentation Ethics Committee of the Potiguar University (0112015) and conducted following the guidelines of the Brazilian Council for Animal Experimentation (CONCEA), which adheres to the National Institute of Health guide for the care and use of laboratory animals. Twenty-five male *Wistar* rats (*Rattus norvegicus*) with 90 days of age and weighing between 273 and 297 g were used in the study. The rats were housed in collective polypropylene cages with controlled temperature (22 ± 1 °C), humidity (50–55%), and a 12 h light–dark cycle, receiving water and diet ad libitum.

### 4.3. Experiment Protocol 

The animals were randomly assigned to five experimental groups: the control group (C, *n* = 5), which received 0.9% saline solution intraperitoneally; the cadmium group (Cd, *n* = 5), which received intraperitoneal administration of cadmium (2 mg/kg); and the test groups, which received oral supplementation by gavage of different parts of the atemoya, forming three distinct groups: T-Se, receiving the seed extract (2 g/kg bw, *n* = 5); T-Pe, receiving the peel extract (2 g/kg bw, *n* = 5); and T-Pu, treated with the pulp extract (2 g/kg bw, *n* = 5). 2 g/g is a safe dose in Annona species [36]. All three test groups received intraperitoneal treatment with cadmium (2 mg/kg) 1 h before treatment with atemoya extracts. Cadmium chloride (CAS number: 10025-69-1) was obtained from Merck (Darmstadt, Germany). Supplementation was carried out for 25 days, during which all groups received a standard Labina^®^ diet and water ad libitum. Biochemical, morphological, and histological tests were conducted on the last day of the experiment.

### 4.4. Activity of Antioxidant Enzymes in Plasma

#### 4.4.1. Catalase (CAT)

The analysis of catalase activity in plasma was conducted using a commercial kit (Cayman Chemical Company, Ann Arbor, MI, USA). This method is based on the enzyme’s reaction with methanol in the presence of hydrogen peroxide (H_2_O_2_). The formaldehyde produced is quantified by a spectrophotometer using a colorimetric reaction. The results of CAT activity were expressed in nmol/min/mL of plasma [37,38].

#### 4.4.2. Superoxide Dismutase (SOD)

Superoxide dismutase activity was measured using the tetrazolium method, which detects superoxide radicals generated by xanthine oxidase and hypoxanthine. This assay quantifies the activity of the three types of SOD (Cu/Zn, Mn, and FeSOD). One unit of SOD is defined as the amount of enzyme required to achieve 50% dismutation of the superoxide radical. The results were expressed as SOD activity in U/mL of plasma [37,38].

#### 4.4.3. Glutathione Peroxidase Activity (GPx)

The glutathione peroxidase activity was evaluated using a method based on the reaction between residual glutathione, after GPx, and 5,5′-dithiobis (2-nitrobenzoic acid) (DTNB). This reaction forms a complex that absorbs maximally at 412 nm [37,38].

### 4.5. Kidney Function Markers

Rats were individually adapted to metabolic cages over three days. On the fourth day, urine was collected over a 24 h period. The volume of urine was measured and kept refrigerated at −20 °C for subsequent analysis. Creatinine levels in the urine were determined using commercial kits, following the manufacturer’s instructions (Bioclin, Belo Horizonte-Minas Gerais, Brazil). In addition, creatinine clearance was calculated based on serum and urine creatinine levels.

### 4.6. Inflammatory Markers in Plasma 

Interleukin-6 (IL-6) and tumor necrosis factor-alpha (TNF-α) were measured using an Enzyme-Linked Immunosorbent Assay (ELISA). The detection limits for IL-6 and TNF-α were 0.7 pg/mL and 0.1 pg/mL, respectively, with intra- and inter-assay coefficients of variation of less than 5%. The analysis was conducted using a SpectraMax automatic optical reader at a wavelength of 570 nm [39].

### 4.7. Measurements of Kidney Morphological Changes

To assess morphological changes resulting from tubular necrosis, kidneys were immersed in a 10% formaldehyde solution at the end of the treatment and subjected to conventional hematoxylin-eosin (HE) staining. Semi-quantitative analyses were performed to evaluate epithelial necrosis, hyperemia, glomerular hypercellularity, increased tubular cells, inflammation, and tubular dilatation. Each parameter was assessed microscopically. Histological sections were stained with HE, and ten cortical areas and five medullary areas were analyzed per animal. The images obtained were evaluated, and quantification was based on a scale of zero to three, representing none, low, moderate, and high alteration, respectively.

### 4.8. Statistical Analyses

Results are expressed as the mean ± SEM. All data were checked for normal distribution using the Shapiro–Wilk normality test. Differences between groups were assessed using one-way ANOVA and the Tukey post hoc test. Statistical significance was considered at *p* < 0.05. Statistical analysis and graphing were performed using GraphPad Prism software (version 8.0, La Jolla, CA, USA).

## 5. Conclusions

This study demonstrated that lyophilized extracts of atemoya derived from seeds, peels, and pulps exhibited significant renoprotective effects against cadmium-induced nephrotoxicity. These extracts restored antioxidant enzyme activities and reduced inflammatory cytokine levels, suggesting potent antioxidant and anti-inflammatory effects. Among the different extracts, the pulp extract showed the highest protective capacity in neutralizing oxidative stress and mitigating renal damage. The results highlight the potential of atemoya fruit in preventing heavy metal-induced kidney damage. The reduction in inflammatory markers and the protection against morphological and functional changes in the kidneys indicate that atemoya could be a promising strategy for the prevention and treatment of nephrotoxicity. Thus, the use of plant extracts rich in phenolic compounds, such as those found in atemoya, may offer significant benefits in renal protection and modulation of inflammatory responses associated with environmental toxin exposure.

## Figures and Tables

**Table 1:** Assessment of weight gain, food intake and diuresis.

Parameters	C	Cd	T-Se	T-Pe	T-Pu	F	*p*-Value
Weight gain (g)	21 ± 3.2	9.5 ± 2.8	8.0 ± 2.9	4.5 ± 2.3	8 ± 2.0	5.56	0.003
Food intake (g/day)	32 ± 2.1	26 ± 3.0	18 ± 3.0	26 ± 3.3	27 ± 2.0	3.37	0.02
Diuresis (ml/day)	10 ± 1.1	9.8 ± 1.0	7.3 ± 2.4	13.3 ± 2.9	10 ± 1.8	1.15	0.35

Group (C) consisted of rats that were not exposed to any treatment (*n* = 5); cadmium group (Cd), the rats received intraperitoneal injections of cadmium (2 mg/kg); T-Se (rats treated with the lyophilized seed extract, *n* = 5); T-Pe (rats treated with the lyophilized bark extract, *n* = 5); T-Pu (rats treated with the lyophilized pulp extract, *n* = 5). All three groups received equal doses of different parts of atemoya (2 g/kg body weight), F, Anova Factor.

**Table 2:** Evaluation of morphological changes seen in histological examinations of rats treated with antioxidant extracts from parts of the fruit exposed to renal injury by cadmium.

Groups	Tubular Cell Enlargement	Inflammation	Tubular Dilation	Necrosis of the Epithelium	Hyperemia	Glomerular Hypercellularity
C	-	-	-	-	-	-
Cd	+++	+++	++	+	+	+++
T-Se	-	-	-	-	-	-
T-Pe	-	-	+	-	-	-
T-Pu	-	-	-	-	+	-

Severity of renal histological changes using the scoring scale: none (-), little (+), moderate (++) and severe (+++).

## Data Availability

The data that support the findings of this study are available from the corresponding author upon reasonable request.
